# Impact of the COVID-19 pandemic on the nursing students' education in a public university in Colombia*

**DOI:** 10.17533/udea.iee.v41n1e11

**Published:** 2023-03-14

**Authors:** Cecilia Leon Palomino, Sandra Catalina Ochoa Marín, Vanessa Restrepo Betancur, Sonia Semenic

**Affiliations:** 1 Nursing master’s student. McGill University, Montreal, Canada. Email: cecilia.leonpalomino@mail.mcgill.ca. McGill University McGill University Montreal Canada cecilia.leonpalomino@mail.mcgill.ca; 2 Nurse, Ph.D. Professor. University of Antioquia, Medellin, Colombia. Email: catalina.ochoa@udea.edu.co Universidad de Antioquia University of Antioquia Medellin Colombia catalina.ochoa@udea.edu.co; 3 Nursing undergraduate student. University of Antioquia, Medellin, Colombia. Email: vanessa.restrepob@udea.edu.co Universidad de Antioquia University of Antioquia Medellin Colombia vanessa.restrepob@udea.edu.co; 4 Nurse, PhD. Associate Professor. McGill University, Montreal, Canada. Email: sonia.semenic@mcgill.ca McGill University McGill University Montreal Canada sonia.semenic@mcgill.ca

**Keywords:** COVID19, students, nursing, education, nursing, qualitative research, COVID19, estudiantes de enfermería, educación en enfermería, investigación cualitativa, COVID19, estudantes de enfermagem, educação em enfermagem, pesquisa qualitativa

## Abstract

**Objective.:**

To explore the impacts of the COVID-19 pandemic on nursing student education in one public university in Medellin, Colombia.

**Methods.:**

This descriptive qualitative study used content analysis to address the following questions: (1) How has the COVID-19 pandemic impacted nursing education at the University of Antioquia? (2) What were the most important challenges experienced by nursing students? (3) What was most supportive for the students during the pandemic? and (4) What were the potential opportunities and lessons learned related to nursing education? Data were collected virtually through individual online interviews with 14 undergraduate nursing students and analysed using qualitative content analysis with constant comparisons.

**Results.:**

Four main categories of findings related to the experience of undergraduate nursing students during the COVID-19 pandemic were identified: (1) transitioning to online learning, (2) managing the digital world, (3) impacts on clinical training, and (4) work-related stressors. Key challenges included home environments that were not conducive to learning, reduced social interactions with peers and faculty, accessing technology required for online education and insufficient preparation for clinical practice. Family members and university-provided resources were important sources of student support. Whereas the pandemic limited opportunities for hands-on clinical training, the shift to online learning allowed for the development of skills related to informational technologies and telehealth.

**Conclusion.:**

Undergraduate students at the University of Antioquia identified significant barriers to learning during the COVID-19 pandemic restrictions and transition to online learning, as well as new opportunities for the development of digital skills among both students and faculty.

## Resumen

**Objetivo.:**

Explorar los impactos de la pandemia de COVID-19 en la formación de estudiantes de enfermería en una universidad pública de Medellín, Colombia.

**Métodos.:**

Este estudio cualitativo descriptivo en el que se utilizaron las siguientes preguntas: (1) ¿Cómo ha impactado la pandemia de COVID-19 en la educación de enfermería en la Universidad de Antioquia? (2) ¿Cuáles fueron los retos más importantes experimentados por los estudiantes de enfermería? (3) ¿Qué fue lo que más apoyó a los estudiantes durante la pandemia? y (4) ¿Cuáles fueron las oportunidades potenciales y las lecciones aprendidas relacionadas con la educación en enfermería? Los datos se recopilaron virtualmente mediante entrevistas individuales en línea con 14 estudiantes de enfermería y se analizaron mediante análisis de contenido cualitativo con comparaciones constantes.

**Resultados.:**

Se identificaron cuatro categorías principales de hallazgos relacionados con la experiencia de los estudiantes universitarios de enfermería durante la pandemia por COVID-19. Estas categorías son (1) transición al aprendizaje en línea, (2) gestión del mundo digital, (3) impactos en la formación clínica y (4) factores de estrés relacionados con el trabajo. Entre los principales retos se encontraban los entornos domésticos poco propicios para el aprendizaje, la reducción de las interacciones sociales con los compañeros y el profesorado, el acceso a la tecnología necesaria para la educación en línea y la preparación insuficiente para la práctica clínica. Los familiares y los recursos proporcionados por la universidad fueron importantes fuentes de apoyo para los estudiantes. Mientras que la pandemia limitó las oportunidades de formación clínica práctica, el cambio a la enseñanza en línea permitió el desarrollo de habilidades relacionadas con las tecnologías de la información y la telesalud.

**Conclusión.:**

Los estudiantes de pregrado de la Universidad de Antioquia identificaron barreras significativas para el aprendizaje durante las restricciones de la pandemia COVID-19 y la transición al aprendizaje en línea, así como nuevas oportunidades para el desarrollo de habilidades digitales tanto entre los estudiantes como entre los profesores.

## Resumo

**Objetivo.:**

Explorar o impacto da pandemia de COVID-19 na formação de estudantes de enfermagem de uma universidade pública de Medellín, Colômbia.

**Métodos.:**

Neste estudo qualitativo descritivo, as seguintes perguntas foram usadas para orientar a entrevista individual virtual síncrona: (1) Como a pandemia de COVID-19 impactou o ensino de enfermagem na Universidade de Antioquia? (2) Quais foram os desafios mais importantes vivenciados pelos estudantes de enfermagem? (3) O que mais apoiou os alunos durante a pandemia? e (4) Quais foram as principais oportunidades e lições aprendidas relacionadas ao ensino de enfermagem? Participaram 14 estudantes de enfermagem, as informações foram analisadas por meio de análise de conteúdo qualitativa com comparações constantes.

**Resultado.:**

Foram identificadas quatro categorias principais descobertas relacionadas à experiência dos universitários de enfermagem durante a pandemia de COVID-19: (1) transição para o aprendizado online, (2) gestão do mundo digital, (3) impactos na formação clínica e (4) fatores de estresse relacionados com o trabalho. Entre os principais desafios estavam ambientes domésticos pouco propícios ao aprendizado, interações sociais reduzidas com colegas e professores, acesso à tecnologia necessária para educação online e preparação insuficiente para a prática clínica. Os familiares e os recursos fornecidos pela universidade foram importantes fontes de apoio para os estudantes. Embora a pandemia tenha limitado as oportunidades de treinamento clínico prático, a mudança para o ensino online permitiu o desenvolvimento de habilidades relacionadas à tecnologia da informação e à telessaúde.

**Conclusão.:**

Os alunos de graduação que participaram da pesquisa identificaram barreiras significativas ao aprendizado durante as restrições da pandemia do COVID-19 e a transição para o aprendizado online, bem como novas oportunidades para o desenvolvimento de habilidades digitais entre alunos e professores.

## Introduction

The unprecedented coronavirus disease 2019 (COVID-19) has disrupted not only the health care system but educational institutions across the world. To prevent the spread of COVID-19, many universities were forced to temporarily cancel their activities and shift from traditional face-to-face to online education. In this context, nursing schools have been challenged to quickly adapt to new educational modes.^1^ Emerging research from both high-income^2^ as well as low- and middle-income countries (LMICs)^3^ demonstrates that nursing students have experienced many educational challenges related to the COVID-19 pandemic, including adapting to online courses, concerns about insufficient clinical training, conflicting emotions during clinical experiences, and fear of contracting COVID-19 or infecting their loved ones^2^^,^^4^^,^^5^ These challenges, in turn, have had a significant impact on nursing students’ psychological and physical wellbeing. Several recent studies have identified increased levels of stress, anxiety, and negative coping behaviours among nursing students.^3^^,^^6^^,^^7^ However, internationally, little is known about nursing students’ perceptions of the pandemic’s positive impacts on their educational experiences, or how they have managed to overcome related challenges. Additionally, there is scant information on the experiences of nursing students during the pandemic in Latin American countries such as Colombia or other LMICs where poverty and income inequality remain a challenge, and access to technologies needed for online education such as computers and Internet may be constrained.^8^

This study aimed to explore the impacts of the COVID-19 pandemic on undergraduate nursing education at a major public university in Medellin, Colombia. The research questions included: (1) How has the COVID-19 pandemic impacted nursing education at the University? (2) What are the most important challenges experienced by undergraduate nursing students? (3) What has been most supportive for the nursing students during the pandemic? and (4) What are the potential opportunities related to nursing education at the university? 

## Methods

### Design and Participants

This research project employed a qualitative descriptive design using content analysis.^9^ Data were collected through virtual in-depth individual interviews with undergraduate nursing students in the university’s Faculty of Nursing. Inclusion criteria for participants were: 1) age 18 and over, 2) was enrolled in the nursing program at the University of Antioquia (UdeA) in Medellín, Colombia in March 2020 (when the initial COVID-19 pandemic restrictions were first put into place and all courses were moved online), 3) at the time of data collection was still a full- or part-time undergraduate student in the Nursing program (or was recently graduated), 4) able to read and speak Spanish, and 5) willing to participate in a video-recorded online interview. 

As recruitment had to be conducted virtually due to pandemic restrictions, a combination of convenience and snowball sampling was used to recruit potential study participants. An invitation letter was emailed to all undergraduate nursing students, obtained through the Faculty of Nursing’s registrar. The letter included a link that directed interested students to the study's electronic information and consent form and a sociodemographic questionnaire, using a secure electronic survey software (Qualtrics). The information and consent form identified the study as a masters’ student project in global health nursing and included the study’s purpose and main research questions. Completion and return of the sociodemographic form implied the participants' informed consent and agreement to participate. The study’s student research assistant and participants were additionally asked to encouraged their peers to participate in the study. Recruitment and interviewing of participants continued until answers to the main interview questions became redundant. No participants withdrew during the study.

### Data Collection

Data collection occurred concurrently with recruitment and took place between November and December 2021. Individual, in-depth interviews via the video conferencing platform Zoom were conducted by the first author (a female registered nurse and Masters’ of Global Health Nursing student) using a semi-structured interview guide with probes. Each student was interviewed once, and all interviews were conducted in Spanish (the interviewer’s first language) at a time and private location of the students’ choosing. The first author received training in interviewing techniques and pilot-tested the interview guide with three Spanish-speaking students prior to participant recruitment. The interview questions addressed participant’s perceptions of the impact of the COVID pandemic on their nursing education; challenges faced during the pandemic; support for students during the pandemic; positive impacts or opportunities created for nursing education during the pandemic as well as recommendations for improving nursing education at the university (Table 1). Interviews lasted between 45 and 60 minutes. In the event of major issues related to internet connectivity, participants were offered the opportunity to close their cameras or continue the interview at a later date. 

**Table 1:** Interview guide

Main Questions	Probes
*In general, how do you feel the COVID-19 pandemic has impacted your nursing education at the UdeA*?	Impacts… on theory classes? your clinical or community hetraining? student life? Interactions with the faculty?
*In your view, what are the most important challenges that you, or other nursing students, have faced during the COVID-19 pandemic?*	Challenges related to your… coursework / theory classes? online learning? clinical or community practicums? ability to study? life as a student? family responsibilities?
*In your opinion, what has been most helpful or supportive for you or other nursing students during the COVID-19 pandemic?*	Supports related to your… coursework / theory classes? online learning? clinical or community practicums? ability to study? life as a student? family responsibilities?
*In your opinion, what have been the positive impacts or opportunities that have been created for nursing education during the COVID-19 pandemic?*	Positive impacts related to… the content of your nursing courses? the clinical or community practicums? virtual/online education?
*What recommendations do you have for improving nursing education at the UdeA during the pandemic, or after?*	Recommendations for… theory courses? clinical or community practicums? support for nursing students? maintaining or improving the quality of the nursing program?

### Data Analysis

Data collection and analyses were conducted concurrently. The audio recordings of the interviews were transcribed verbatim by a Colombian student research assistant and analyzed using qualitative content analysis with constant comparisons. Transcript were not returned to the participants. Transcript data were coded in their Spanish using both inductive and deductive approaches.^11^ First, a broad general framework based on the main categories of questions (i.e., challenges, supports, opportunities, recommendations) was used to organize the analysis. The transcripts were then read and reread to highlight words or phrases that reflected the thoughts and issues corresponding to each research question. These highlighted words and phrases were compared, assigned codes, and merged across participants to generate a preliminary coding frame. The preliminary framework was refined with each subsequent transcript by examining similarities and differences, as well as new ideas that generated additional codes. The emerging codes were then grouped into main categories and sub-categories. Microsoft Word and Microsoft Excel programs were used to store, organize, and facilitate the data analysis. First transcripts were initially coded separately by the first author and a co-author (nurse, PhD) and emerging categories were compared; any differences in coding were resolved by discussion. To enhance trustworthiness of the analysis, regular meetings were held with the first author and two co-authors with PhDs in Nursing throughout the analysis phase to discuss the evolving codes and category framework. To further enhance trustworthiness, the first author kept field notes throughout the data analysis process to record personal reflections on the interviews, and to keep a record of analytical decisions made during the coding and categorization process. 

### Ethical Issues

Ethics approval was obtained from the Institutional Review Board (IRB) of the McGill Faculty of Medicine and Health Sciences (IRB review number: A05-B45-21B, 14/05/21), as well as the Research Ethics Committee of the Faculty of Nursing, University of Antioquia (Acta number CEI-FE 2021-19, 20/02/21). All participants were informed that their participation was strictly voluntary and they could withdraw their participation at any time during the interviews; their identity would remain anonymous by assigning a numerical code to their interview data and any quotes used in the final research reports; the study data would be kept in password-protected electronic files; and only members of the research team would have access to the interview data. 

## Results

A total of fourteen participants were recruited and completed the online interview. The demographic characteristics of the participants are presented in Table 2. Most were in their first year of the program in March 2020, when pandemic restrictions required all classes to be shifted online. At the time of the interviews in the autumn of 2021, all courses and clinical placements were still being conducted virtually, and only final year students were able to attend their clinical practicums in-person. All participants were full-time students, and the majority (*n*=9) were female, three of whom were single mothers. Six students were between 18 and 21 years old, while three were at least 30 years old. All participants identified as low-income, belonging to the the three lowest income brackets from Colombia’s socioeconomic strata of 1(very low) to 6 (high). Five participants were working part-time while in school, including two who worked in a healthcare facility with COVID-19 patients. Despite multiple e-mailed invitations during the participant recruitment phase, no nursing students who were in their 5th, 6th, or 8^th^ (final) semester in March 2020 volunteered their participation.

**Table 2:** Participant socio-demographics (*n*=14)

Variables	*n*
Gender	
Male	5
Female	9
Age (years)	
18-21	6
22-25	2
26-29	3
≥30	3
Social strata	
Very low income	1
Low income	4
Medium low income	9
Semester	
1	2
2	8
3	2
4	1
7	1
Working status	
Working in health care	2
Working in other fields	3
Currently not working	9
Number of people living in the student’s household	
3	5
4	5
6	4
Has dependent children	
Yes	3
No	11

The transcribed interview data were coded and organized into four over-arching categories: 1) transition to online learning; 2) managing the digital world; 3) impact on clinical training, and 4) work stressors.

### Transitioning to online learning

#### Adapting to virtual classes

When all face-to-face classes were suspended, students had to quickly adapt to a new teaching methodology on online learning. Several participants perceived an increase in workload, requiring more time in front of the computer with less free time for themselves. Some explained that professors were delivering more content at a faster pace during online classes, knowing that lectures were recorded and could be reviewed. Others noted that professors were using the new online learning platforms to assign more homework and extra readings and that some teachers believed that remote learning was easier for students as they had “more time [available when studying] online” (P9). On the other hand, participants noted that some professors had *a methodology very much linked to traditional teaching [...] and who wanted everything to be exactly like [in] class* (P7) without considering the possible difficulties encountered with online learning, such as Internet access problems or lack of electricity. 

Another challenge expressed by numerous participants was that online class hours were extended, and some professors did not respect breaks between courses, contributing to feelings of being *glued to a screen all day* (P3), overloaded with information, and prevented from *clearing their minds* (P6) before changing courses. Participants also noted that it was embarrassing to ask questions during class, as all attention was focused on someone who turned on their microphone/camera or typed a question in the chat, which decreased student participation. Two participants additionally expressed that the use of the camera raised privacy concerns related to *feeling observed* (P12) or that their *privacy was being invaded* (P13). One participant described a feeling of being disconnected from classes, as taking notes was a strategy for being linked to class and this was no longer necessary as all the materials covered in class (e.g., power point presentations, PDFs) were sent directly to the students. Note-taking was seen by some participants as an essential component of their learning, as it facilitated internalization of knowledge and preparation for exams. However, the delivery of lectures using digital pedagogical tools and the speed at which the course content was delivered made note-taking more difficult. The lectures for synchronous classes were streamed live and recorded for the students. However, some participants felt the recorded lectures left them with a lot of *doubts and unanswered questions* (P6), in addition to the extra time required to transcribe the recordings into class notes. Completing virtual exams were an additional challenge mentioned by participants, due to difficulty concentrating at home and technological issues with connecting to the Internet or poor screen resolution. 

Despite a variety of challenges adapting to online classes, participants described new opportunities offered by the transition. For example, some noted a greater interest in online searching to better understand topics covered in class: *a positive impact [of the pandemic is that it] has increased people's options to search for information, so as not to simply stay with what is printed in the book* (P7). The move to online learning also provided participants with *easy access* (P3) to virtual conferences or seminars from the Nursing as well as other university faculties, allowing them to acquire *new learning perspectives* (P2). Also, six participants felt that the class recordings were useful as they allowed them to review the classes as many times as needed to capture any information missed and access information on complex concepts that required more memorization. Class recordings were also perceived as very practical for working students who could not attend classes synchronously or for those who had Internet issues. Furthermore, saving time and money were a positive aspect of online learning raised by five participants, as they did not have to pay for transportation and food, waste time in traffic, or *have to get up very early in the morning to get to a classroom* (P4). Two participants also discussed how online education helped them learn to better manage their time and to balance academic, family, and work responsibilities. 

To facilitate the transition to online learning, many participants recommended more training for professors to optimize their online teaching skills, for example, the use of other digital learning spaces to make classes less monotonous and not *[look] at slides all the time* (P1). Half of the participants expressed the need to return to face-to-face classes as soon as possible to foster social interaction and enhance their learning and hands-on training, whereas others recommended maintaining a hybrid education to keep the positive aspects of online education (i.e., saving money/time and access to class recordings) while still benefiting from face-to-face classes.

#### Managing the study environment

With the closure of face-to-face classes and lockdowns, most students had to attend classes from their home or workplace. Difficulty concentrating at home was a frequently mentioned challenge given multiple distractions including *noises in the house* (P1) (e.g., pets and family) or *noises [coming] from the street* (e.g., street vendors, cars) (P7). Many of the participants lived in a small home with multiple family members who frequently distracted them: *My grandmother didn't really understand [the virtual world and] if I was in the bedroom [studying], she would come in, give me the baby, [and] talk on the phone next to me* (P3). Small in-person class blocks were replaced by longer online classes of two hours or more, further compromising concentration. It was difficult for participants to find a conducive study environment while sharing space with other family members who were also confined and working/studying from home, requiring participants to *invest a lot more energy and time to concentrate* (P8). Participants often had to negotiate moments of silence with family members during online classes or evaluations, or were resigned to studying in their rooms, on their beds. Participants also described being distracted by social media or the web when accessing their cell phones or computers for classes, further dividing their attention: *Notifications would get to [my] cell phone and just out of curiosity [I] would open [it] and stay [on it]* (P8).

Another difficulty mentioned by some participants was that their families did not recognize their role as students. For example, one mentioned: *It was difficult [...] trying to make [my family] understand that if I’m on a computer I’m not playing [or] entertaining myself [...] they think that because you’re at home you’re available for everything* (P6). Some participants had to assume new responsibilities when studying at home such as meal preparation, household chores or caring for an older adult, which were primarily handled by their parents prior to the pandemic. Those who had children had the additional challenges of balancing studies with parental responsibilities, as they had to assist their children with online classes and schoolwork while attending to their own, in addition to responding to their children’s other needs. Participants highlighted the help of family members (e.g., parents and siblings) with childcare, household chores, and meal preparation as an important support for their academic activities, as well as the willingness of family members to *adapt certain spaces in the home* (P1) to create a more conducive learning environment. 

#### Reduced social interactions with faculty and peers

Decreased social interactions with faculty and students was one of the most significant impacts experienced by participants related to online education: *virtuality distanced us a lot [because] in the nursing faculty, [we] considered ourselves very familiar, so [we were] always in touch, kissing and hugging* (P1). Participants reported that the way they used to interact with professors and understand things were significantly transformed with online learning. Whereas in the classroom professors were able to maintain visual contact with their students and recognize when something was unclear to them, this was not possible with virtual classes because the camera was often not used. Participants also mentioned that it was difficult to interact with professors and seek help or even ask questions without having established a previous relationship with them. COVID-19 restrictive measures and the closure of the university further prevented students from frequenting places where they used to study together and share their learning (e.g., the library, the school hallways during breaks or their classmates' homes), leading to a *decrease in knowledge sharing* (P6) and peer learning. In addition, as communication between students was limited to WhatsApp calls or text messages, group assignments for online courses were *more complicated to organize* (P6), compared to when the university had study spaces. Participants described numerous impacts on their mental health as they were confined for so long with little social interaction and higher stress levels due to increased academic and family responsibilities. Some reported experiencing anxiety and depressive symptoms such as sadness, lack of motivation and sleep problems.

University-provided group psychological counselling was a critical source of support frequently mentioned by participants, as these spaces allowed students to *talk about emotional situations related to [their] personal or academic environment* (P6) and engage in virtual recreational activities (e.g., applying make-up, drawing, cooking classes) that offered opportunities to interact with other students, make friends and create new connections. Participants also expressed that their university provided holistic and continuous support by being attentive to their needs. For example, a 24-hour telephone helpline service was offered for students who were experiencing a "crisis moment" with psychological follow-up if needed. However, one participant felt that this psychological service should have been more widely promoted with more guidance for students on how to access it. Additional strategies mentioned by participants for coping with online learning and related stressors included exercising, seeking spiritual or religious refuge, and reaching out to friends. 

### Managing the Digital World

#### Access to technology

Not all the students interviewed had a computer or Internet connection. Participants who lived in rural areas or had to share the Internet at home with other family members were challenged by unstable network connections. Others had to share the only computer at home with their siblings or children, forcing them to connect to classes from their cell phones: *[When the laptop was unavailable] I used my cell phone to connect to class, it was uncomfortable… I couldn't see well; it was very difficult* (P8). Online education was perceived by some participants as a *privilege* (P2) as not everyone had *the same possibilities* (P4) to access to technology. Participants recognized the emergence of digital inequalities during the pandemic, and some offered support to their peers by lending them their computers or giving them access to Internet connections. Others obtained Internet access by connecting to a family member's cell phone Internet or using their neighbour/friend's WIFI. Students on the university’s student council played a key role in advocating for the provision of technological resources and financial aid to ensure that all students were equipped for online learning. The understanding and flexibility of professors who were open to listen to student difficulties with virtual learning was highly appreciated by the participants.

### Digital skills

Lack of skills to navigate through the different digital platforms used for online classes was highlighted by some participants, as initially *very few were familiar with the use of Zoom and Google meet* (P7). One participant also described a lack of technological skills with online education among nursing faculty, especially by older professors who had more difficulties managing digital platforms. On the other hand, the adoption of new digital skills by students experiencing telehealth as part of their clinical training was perceived as a positive element in their academic lives that allowed greater access to information and stimulated their creativity: *The virtuality helped us develop some skills and knowledge [for patient education], which involved searching for very creative websites, using slides and videos* (P1). Another positive aspect of the telehealth experience perceived by participants was that technology could be used to reach people far from health care providers, allowing them greater access to health services and health education.

### Impact on Clinical Training

#### Technical procedures

When the pandemic began, in-person labs were reduced or cancelled, and clinical practicums were cancelled or limited exclusively to final year students. This left participants with a lot of *doubts* and a feeling of *insufficient knowledge to move on to the next course* (P1). Virtual labs consisted of viewing different procedures or technical concepts through videos or lab guides and were not seen as equal to in-person labs. As result, participants felt less prepared to treat patients face-to-face, which generated *fears and more pressure* (P2) about the clinical environment. In addition, the COVID-19 pandemic limited participation in clinical practicums since many health services were closed to students, reducing opportunities for learning in different clinical settings. Participants recommended that the university extend the hours of practice of in-person labs, which were considered essential for the development of their technical and clinical skills. 

#### Communication skills

The development of communication skills was also reported to be affected by the absence of face-to-face contact with patients in the clinical setting: *[Virtuality] affects the way you talk to the person and determine if the person understands what you are saying, so I think it's inevitable [that virtuality] affects [communication skills]* (P6). Although community health practicums were held virtually via telehealth participants did not feel this represented the *real scenario* (P6) where patient care is delivered face-to-face and noted several barriers to communicating with community clients online. For example, it was it difficult to capture the attention and interact virtually with children when connecting with younger clients, or to establish a therapeutic relationship with clients virtually via the camera and microphone, which made it difficult for people to open up and ask questions.

### Work-Related Stressors

#### Balancing work and school

Some participants had to work during the pandemic in different occupations (e.g., opening an Internet business, working in a pharmacy, making deliveries) due to the closure of non-essential businesses and the loss of many jobs. Some had to work to help their parents cover additional household expenses generated by confinement during the pandemic (e.g., increased electricity bills), and the single parents had to provide for their dependent children. One single-parent participant with two daughters under 18 years of age reported that she had to *work more and spend less time at home* (P7) during the pandemic. The two participants who worked as nursing assistants reported difficult working conditions and high levels of stress caring for patients with COVID and bereaving the loss of both patients and co-workers, requiring one to seek psychological support at work. They had the additional challenges of trying to attend their classes from work when they were needed for patient care. Similar challenges to concentration were experienced by other participants who attended classes while working: *I was listening to the class without really paying attention* (P7). Some participants also reported a decline in their academic performance or that of their peers, most notably among students who worked and/or perceived an increased study load.

#### Fear of infection

The fear of getting infected with the COVID-19 virus was particularly significant for one participant who worked in a COVID-19 unit, whose greatest concern was infecting her family, as their health *came first* for her (P7). The participant noted that one of the main challenges as a nurse during the pandemic was feeling rejected by family members who were afraid of being infected by her. On the other hand, participants who were able to attend clinical practicums during the pandemic felt the university supported them by providing the necessary personal protective equipment (e.g., surgical mask, #95 mask) as well as access to COVID-19 vaccines, which helped calmed fears and insecurities related to COVID-19 infection. 

## Discussion

This study explored how the COVID-19 pandemic impacted the learning experiences of predominately low-income nursing students at one public university in Medellin, Colombia. Consistent with other recent findings,^11^^-^^14^ this study found that the shift to online learning reduced important opportunities for social interaction between students and faculty, including the ability to work together, create study groups, exchange notes, and easily ask questions to faculty after class. Similar to Langegård *et al*. ^11^, this study found that face-to-face interactions allowed nursing students to have a deeper understanding of their course material and was considered to be beneficial to the progression of their learning. Hamadeh Kerbage *et al*.^15^ also found that although nursing students attempted to compensate for face-to-face interaction through social media, core interactions considered valuable to students were still missed. These findings highlight the importance of institutional efforts to create and promote alternative ways for students to socialize. This may be particularly important in cultures such as that of Colombia, where close relationships between students and faculty are common and highly valued. Therefore, to improve students' perceived social presence^16^ and foster an ongoing sense of community,^12^ there is a need to implement more engaging activities (e.g., blogs, break rooms, video message sharing) in the online setting to actively involve students with their peers and faculty. 

Most of the participants in this study found studying at home to be challenging, as home was not perceived as a conducive environment for their learning due to multiple distractions and limited space. Consistent with the findings of Wallace et al.^12^^) )^ participants in our study experienced major distractions in their home environment such as noise and the demands of family members, making it more difficult to transition to online education. Wallace *et al*.^12^ similarly found that changes in family roles and dynamics following confinement generated stress for nursing students as they faced greater responsibilities in addition to their academic workload (e.g., caring for younger siblings and/or an elderly relative). As found in previous studies, ^(^^12^^,^^17^ the difficulty of studying from home appeared to be particularly great for students who were also parents, as they had to meet the needs of their children who were also at home and take on different household responsibilities. Therefore, educators need to be flexible and consider these additional burdens for student-parents to facilitate the online learning transition.

Consistent with previous studies,^12^^,^^13^^,^^17^^,^^18^ Internet connectivity issues were reported as a major challenge by participants in this study. This challenge was greater for participants who returned to their home away from Medellin, where Internet access was reported to be more difficult.^19^ An interesting finding from this study was that lack of equipment and insufficient Internet bandwidth at home forced some students to rely on their cell phones to access their courses. This is an important consideration that faculty members should consider when designing online courses, in order to create a mobile-friendly online learning environment. Such challenges to online education have created inequities in educational access in developing countries that academic institutions must recognize.^20^ As found in our study, the provision of technological resources (e.g., computers, Internet access) and financial assistance provided by the university was crucial for ensuring equitable access to online education and facilitating program completion.

Our findings that students felt unprepared for their practical experiences due to insufficient in-person clinical training have also been reported by several previous studies.^13^^,^^20^^-^^22^ Gallego-Gómez *et al.*^21^ found that the unavailability or insufficiency of clinical training experiences generated many uncertainties and fears in nursing students, including how their future careers may be affected by online education and the cancellation of clinical placements. Agu *et al.* ^(^^20^ similarly found that nursing students felt that online education reduced the opportunity for nursing students to develop the skills (e.g., hands on practice, communication skills) necessary for clinical practice. However, Orin^23^ noted that the challenge of providing relevant clinical experiences has resulted in an increased use of simulation, telehealth and virtual reality. In our study, replacing in-person community health experiences with virtual encounters with clients were appreciated by the students as they created opportunities to develop new skills with telehealth. Therefore, educators need to explore other possible means of acquiring clinical skills (e.g., via simulations) and ensure that students have the technological resources necessary to help build technical skills virtually. 

Study participants working with COVID-19 patients expressed concern about the possibility of contracting the virus, as they feared infecting their loved ones. Several other studies have identified feelings of fear and guilt among nursing students related to infecting a loved one with COVID-19.^6^^,^^21^^,^^24^ Martin-Delgado *et al.*^25^ found that fear of infection was exacerbated by the lack of personal protective equipment (PPE) in developing countries including Colombia where resources are constrained, putting health care workers at increased risk of COVID-19. Our study showed that, in general, fear of infection was greater among students who worked with COVID-19 patients and lived in multigenerational households, as is often the case in LMICs. For example, Kochuvilayil *et al.* ^(^^3^ found that fear of infection was higher among Indian nursing students than among Australian nursing students, which they attributed to living in multigenerational households and cultural influences. More research is needed on the psychological ramifications of this fear of infection among nursing students, which may be more prevalent in LMICs.

Similar to the findings of Gallego-Gómez *et al*.,^21^ students in this study reported experiencing financial concerns due to job loss and increased household expenses caused by extended lockdowns. Nonetheless, in our study, participants found the online courses to be an opportunity to save money associated with transportation and food costs associated with traditional face-to-face education. Therefore, educational administrators must recognize the additional financial stressors that students, particularly those in LMICs, need to go through in order to find financial solutions that will support the continuity of their online education and ensure the future nursing workforce.

Study Limitations. The study sample was from a single public university in Colombia, so the findings may not be representative of nursing students' experiences in other settings. Findings are also limited to the experiences of undergraduate students, therefore future research should include students at higher levels of the nursing program, such as graduate and doctoral students. The small sample size and convenience sampling may not have captured the full range of challenges and opportunities faced by students from diverse backgrounds, such as those who are parents or experience disabilities, or those who are more affluent and/or attending private universities. Finally, Internet connectivity problems occurred at certain points during the online interviews, making it more difficult to communicate with participants and disrupting the discussion flow. Turning off the participants' cameras helped to improve the sound quality but limited the ability to observe participants for non-verbal communication cues. 

## Conclusion

To the best of our knowledge, this is the first study conducted in Colombia to explore nursing students’ learning experiences during the COVID-19 pandemic. Findings revealed that key challenges to nursing education at the University of Antioquia imposed by the COVID-19 pandemic included having to manage studies from home in the presence of limited privacy and multiple distractors, a reduction in social interactions with peers and faculty that were considered necessary for the progression of learning, inequities in accessing technological resources required for online education and insufficient preparation for clinical practice due to the cancellation of clinical placements and limited exposure to direct patient care. However, the COVID-19 restrictions provided new opportunities for learning such as flexible online classes and the development of digital skills, and revealed the critical role of the university in providing psychological and technological support to undergraduate nursing students during the global pandemic.
